# How many neurons does it take to tell left from right?

**DOI:** 10.1111/ejn.15722

**Published:** 2022-06-07

**Authors:** Alid Al‐Asmar, Alfonso Pérez‐Escudero

**Affiliations:** ^1^ Centre de Recherches sur la Cognition Animale (UMR5169), Centre de Biologie Intégrative, CNRS, UPS, Université de Toulouse Toulouse France

Can *Caenorhabditis elegans*, a blind 1‐mm long nematode, learn the position of food in a T‐shaped maze? One would expect that it cannot. In a typical T‐maze learning experiment, the individual is first trained with a reward at one of the two sides of the T. After the training, the individual is placed again in the maze, which is now devoid of any cues and has no reward, and must remember whether the correct corridor was on the right or on the left. This task requires not only memory, but also spatial awareness: The individual must be able to recognize the intersection and know its orientation with respect to it; only then can it use the learned information about the side of the reward. *C. elegans* has a 302‐neuron nervous system and navigates mostly reacting to chemical, thermal and mechanical stimuli. It lacks vision, so it would be exceedingly difficult for it to tell its position and orientation inside a maze—even to tell whether it is inside a maze.

However, a recent paper showed that *C. elegans* learns how to solve a T‐maze (Gourgou et al., 2021). The experiment had a single training trial in which a worm was placed in the maze with a patch of food either on the left or the right branch. Worms that successfully reached the food were transferred to a clean T‐maze, without food or any other chemical cue, and more than 70% of them chose the correct branch. Following the same protocol without food in the training trial led to a 50:50 choice in the test trial, which shows that the result involves food‐mediated learning. This memory was short‐lived (less than 5 min), and abundant controls ruled out the effect of unintended cues (such as odors spreading in the lab, or even the earth's magnetic field).

This result is of primal importance, not only for *C. elegans* but for a large family of simple organisms. Despite the growing evidence that simple organisms—even those without a nervous system—have advanced cognitive abilities, including memory and complex navigation (Dussutour, 2021), this is probably the first time that a moving organism lacking vision and hearing has been observed to remember the position of a food item, and to be able to retrieve this information after being transferred to a new unmarked setup.

How can *C. elegans* accomplish this? Sakelaris et al. (2022) explore this question by building a model of the subcircuit responsible for this type of learning in *C. elegans*. Using the actual connectivity of *C. elegans'* neural network (which is known at the level of individual neurons), Sakelaris et al. modeled the subcircuit responsible for this type of learning. While they use a simplified version of the actual neural network, their model exposes the role of individual neurons while at the same time incorporating realistic dynamics about their activity and interactions. Their model shows that the reinforcement of a single connection between the dorsal and ventral motor subcircuits is enough to produce the observed results (Figure 1a).

**FIGURE 1:**
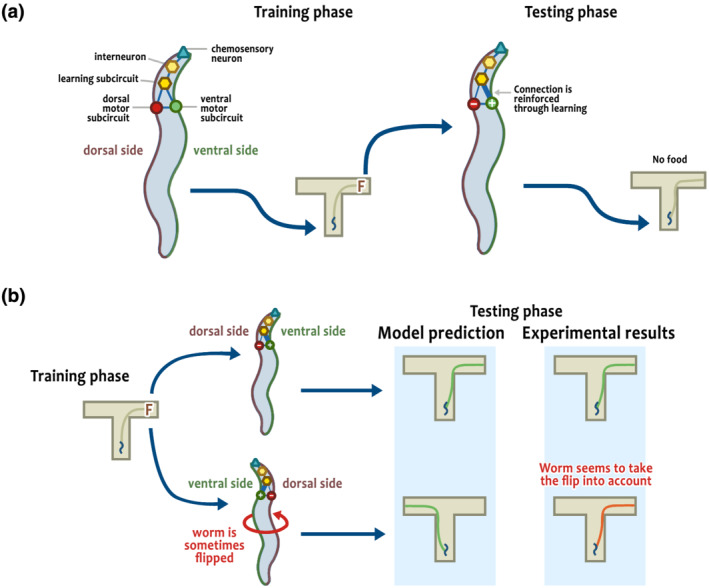
(a) *C. elegans* (seen from the top), with a simplified schematic of the neural network proposed by Sakelaris et al. (2022), before the training (left) and after the training (right). The network comprises a chemosensory neuron (triangle) and two motor circuits (circles), which interact through interneurons (hexagons). *C. elegans* is initially placed on a T‐maze with food (training), then on one without food (test). (b) Comparison between a worm that maintains the same orientation between training and test (top) and a worm that is flipped (bottom). The model predicts the flip to change the side chosen in the testing phase (blue background, left), but the flip had no effect in the experiments (blue background, right)

How can a single connection produce spatial learning? It turns out that, rather than recognizing the intersection and making a discrete choice, worms have a small but constant turning bias towards the correct side throughout the whole test trial. This tendency first makes them more likely to reach the wall of the initial corridor and progress next to it, before they enter in the correct arm of the T‐maze (Gourgou et al., 2021; Sakelaris et al., 2022). This turning bias allows *C. elegans* to reach the correct side of the maze despite its very limited spatial awareness.

What is the adaptive value of this turning bias outside the laboratory? One possibility is suggested by the short timescale of the memory: Worms often wander around the edge of a food patch, entering and exiting repeatedly. Over a short enough timescale the food remains on the same side, so a learned turning bias would increase the chance of reaching the goal (here, food). Another possibility is that it has no adaptive value, being a side‐effect of other functions. It is well established that *C. elegans* can learn to associate food to different cues, such as a given substance or temperature (Ardiel & Rankin, 2010). Given that synapses active before a reward tend to be reinforced, and that the same neurons are reused for different behaviors in *C. elegans'* tiny nervous system, a slight turning bias might be generated as a side‐effect of other types of learning—even if this bias has no measurable effect in natural conditions.

But an intriguing piece of the puzzle remains to be solved, and suggests that this behavior may not be a side‐effect after all. When crawling on an agar surface, *C. elegans* lies on its side. Therefore, when we talk about turning to the right or to the left, from the worm's reference this corresponds to its ventral or dorsal side (just like a human lying on their side can bend towards the belly or towards the back). Accordingly, the model proposed by Sakelaris et al. describes a ventral/dorsal turning bias, and for this model to work worms must lie on the same side of their bodies during both phases of the experiment: If the worm flips in between, the learned bias will lead it to the wrong side (Figure 1b). It turns out that half of the worms did flip between the two phases of the experiment, yet this did not prevent them from reaching the correct side (Gourgou et al., 2021). Therefore, it seems that *C. elegans* has the sensory and neural machinery needed to detect on what side it is lying, and to correct for the flip of its body orientation in order to transform a dorsal/ventral turning bias into a true right/left one. This correction might partially explain why proprioception is necessary for this type of learning (Gourgou et al., 2021).

The work by Sakelaris et al. (2022) is an invaluable contribution to solving these questions. Their model is realistic enough to provide predictions about the role of specific neurons, while simple enough to remain tractable. It provides a unique insight on the minimal neural apparatus required for acquiring a dorsal‐ventral asymmetry, and opens the question of the role that such an asymmetry may play in navigation strategies in *C. elegans*.

## CONFLICT OF INTEREST

We declare no conflict of interests.

### PEER REVIEW

The peer review history for this article is available at https://publons.com/publon/10.1111/ejn.15722.
